# *Wohlfahrtiimonas chitiniclastica*: current insights and complementary review from Chinese cases

**DOI:** 10.1128/aem.00965-24

**Published:** 2024-11-27

**Authors:** Qin Yuan, Cheng Peng, Xin-Lin Sun, Zi-Chun Nie, Yi-Wen Zhang, Ying-Ye Miao

**Affiliations:** 1 Clinical Laboratory, Guiqian International Hospital 701575 , Guiyang, China; UMR Processus Infectieux en Milieu Insulaire Tropical, Ste. Clotilde, France

**Keywords:** *Wohlfahrtiimonas chitiniclastica*, matrix-assisted laser desorption/ionization time-of-flight mass spectrometry, 16S rRNA sequencing, antibiotic resistance

## Abstract

*Wohlfahrtiimonas chitiniclastica* is an emerging zoonotic pathogen associated with bacteremia, myiasis, and soft tissue infections. It is insufficiently identified and underestimated due to reasons, such as shortcomings of the traditional identification techniques and language barriers in local case reports from different regions. In this review, we summarize the currently available literature. In particular, we added previously overlooked cases from Chinese and other medical communities. The clinical characteristics, identification, and treatment of *W. chitiniclastica* are discussed. This work provides a complete review of the previous work including cases from human, animal, and other sources.

## INTRODUCTION

*Wohlfahrtiimonas chitiniclastica*, an oxidase-positive, catalase-positive, Gram-negative, non-motile, and non-spore-forming bacillus, was isolated for the first time from the third-stage larvae of the obligate ectoparasitic fly *Wohlfahrtia magnifica* by Tόth et al*.* in 2008 (1). It belongs to the class *Gammaproteobacteria* (1–3). The genus *Wohlfahrtiimonas* consists of three species: *W. chitiniclastica* (1), *W. larvae* (4), and *W. populi* (5). Recently, Montecillo has reclassified the genera *Ignatzschineria* and *Wohlfahrtiimonas* as members of a novel family *Ignatzschineriaceae*, within the order *Cardiobacteriales* (6). As for the characteristic, *W. chitiniclastica* expresses strong chitinase activity, which may play a role in metamorphosis of its host fly (7). The clinical morphology of *W. chitiniclastica* colonies is entire, convex, smooth, clearly spreading and glistening (8, 9). *W. chitiniclastica* was described as a strictly aerobic bacterium in most reports. However, Ahmad et al*.* (3) have revealed that *W. chitiniclastica* is facultatively anaerobic. Nogi et al*.* (10) and Chavez et al*.* (11) have also reported the anaerobic growing of *W. chitiniclastica*.

Over the past decade, *W. chitiniclastica* has been recognized as an emerging zoonotic pathogen with increasing reports in both humans and animals worldwide. It was reported to be commonly associated with bacteremia, myiasis, and soft tissue infections. Some reports have shown that *W. chitiniclastica* might be misidentified by the traditional identification methods. On the other hand, the local case reports often lack the international attention due to the language barriers (12–17). Therefore, it can be assumed that *W. chitiniclastica* was not fully detected and underestimated (2). In this work, we give a review of *W. chitiniclastica,* including the most complete cases recorded from 2008 to the present.

## LITERATURE REVIEW

**Fig 1:**
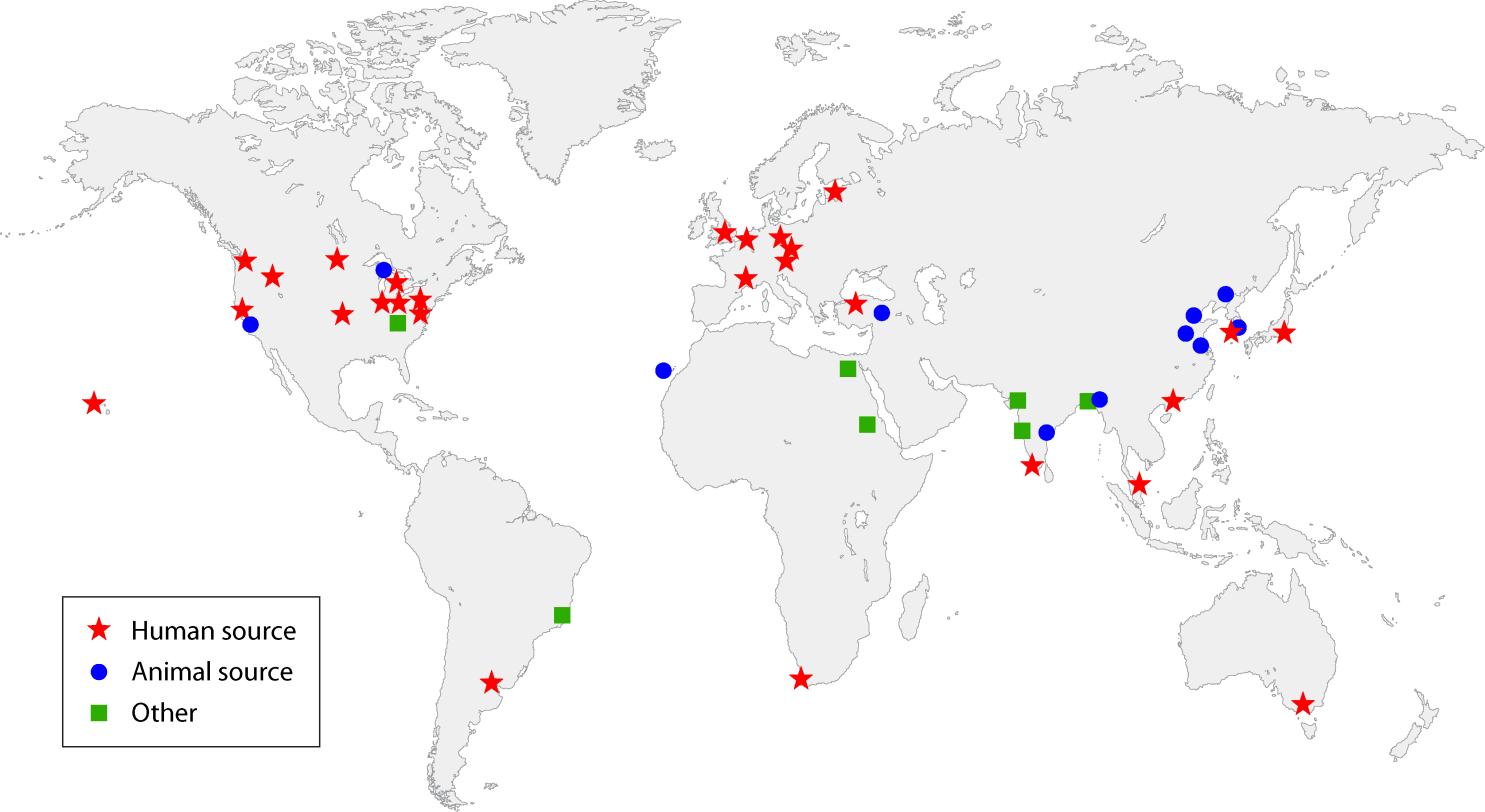
Geographical distribution of *W. chitiniclastica* reports.

*W. chitiniclastica* is an emerging zoonotic pathogen. So far, human cases account for the vast majority of reports (2). In Tables 1 and 2 , we give the brief overviews of the documented cases from human, animal, and other sources since 2008, respectively, in which we add previously unnoticed cases from the Chinese medical community (12–16) and other regions (17). *W. chitiniclastica* was reported to be present on major continents inhabited by humans, with a wide geographical distribution, as shown in Fig. 1. It has been most frequently reported in developed Europe and the United States, and less frequently elsewhere. It is believed that this is due to the lack of technical support in developing and backward regions or to the fact that some case reports are ignored for a number of reasons, such as language barriers. On the other hand, the comprehensive public database of nucleotide sequences GenBank has contained over a hundred global records related to *W. chitiniclastica*, far exceeding the number of formally published case reports. These suggest that *W. chitiniclastica* may not be of high specifically geographical dependence and is underestimated.

**TABLE 1:** Overview of human cases of *W. chitiniclastica*

Case	Year	Region	Age	Gender	Description	Maggot detection	Literature
1	2009	Marseille, France	60	Female	Homeless, alcoholism, ulcers	Yes	(7)
2	2011	Buenos Aires, Argentina	70	Male	Homeless, alcoholism, smoking, occlusive peripheral arteriopathy of the lower limbs, fulminant sepsis (fatal)	No	(9)
3	2015	Tartu, Estonia	64	Male	Alcoholism, gangrene in the distal part of the legs and amputations of the toes, soft tissue and bone infection	No	(18)
4	2015	Guildford, UK	82	Female	Recurrent falls, hypertension, chronic kidney disease, ischemic heart disease, hypercholesterolemia, osteoarthritis, bacteremia-associated myiasis	Yes	(19)
5	2015	Trivandrum, India	43	Male	Alcoholism, smoking, ulcers, cellulitis, osteomyelitis at plantar aspect of right lower limb, gangrene at the right toe, diabetes	-^*a*^	(20)
6	2015	Salt Lake City, USA	26	Male	Morbid obesity, ulcers, cellulitis	-	(21)
7	2015	South Africa	-	-	Soft tissue infection	-	(22, 23)
8	2016	Cape Town, South Africa	17	Male	Degloving injury of the right upper arm and shoulder, soft tissue infection	No	(23)
9	2016	Honolulu, USA	72	Male	Stroke (fatal)	Yes	(10)
10	2016	Honolulu, USA	69	Female	Homeless, right hemiparesis from a ruptured cerebral aneurysm, multiple purulent decubitus ulcers	No	(10)
11	2017	Kubang Kerian, Malaysia	47	Female	Rectosigmoid colon adenocarcinoma metastasized to the lungs and liver, immunosuppression (fatal)	No	(24)
12	2017	Columbus, USA	41	Female	Smoking, paraplegia, ulcers (fatal)	No	(11)
13	2017	Dresden, Germany	79	Male	Diabetes mellitus, coronary heart disease, chronic renal failure, venous insufficiency, progressive ulcerative disease	No	(2, 25)
14	2017	Dresden, Germany	43	Male	Homeless, alcoholism, diabetic foot, ulcerative disease	No	(2, 25)
15	2017	Dresden, Germany	78	Female	Diabetic foot, severe obesity, chronic venous insufficiency, arterial hypertension, chronic heart failure NYHA II, progressive ulcerative disease	No	(2, 25)
16	2017	Dresden, Germany	71	Male	Diabetic foot, deep vein thrombosis, leg ulcers, alcohol intoxication	No	(2, 25)
17	2018	Washington, USA	57	Male	Wet gangrene of the right ankle, myiasis below waist (fatal)	Yes	(26)
18	2018	Tokyo, Japan	75	Male	Squamous cell carcinoma, chronic wounds with maggots	Yes	(27)
19	2018	Indianapolis, USA	37	Male	Myiasis of lower left extremity, ulceration of the toes and lateral calf, sepsis	Yes	(28)
20	2019	Lexington, USA	63	Male	Alcoholism, smoking, necrotic foot ulcer, cirrhosis (fatal)	Yes	(8)
21	2019	Lexington, USA	87	Female	Homeless, myiasis of a left lower extremity wound	Yes	(8)
22	2019	Melbourne, Australia	54	Male	Alcoholism, chronic inflammatory demyelinating polyneuropathy, hereditary hemochromatosis, partial thickness burn wound, sepsis	Yes	(29)
23	2019	Santander, Spain	66	Female	Incised-contused wound in the left pretibial area, ulcers	No	(17)
24	2020	Harrisburg, USA	82	Male	Mitral stenosis, peripheral vascular disease	Yes	(30)
25	2021	Fargo, USA	70	Male	Ulcerative and infiltrative soft tissue lesion in the left temple	Yes	(31)
26	2021	Stanford, USA	50	Female	Homeless, substance abuse, maggot-infested skin ulcers, basal cell carcinoma, cellulitis	Yes	(32)
27	2021	Brno, Czech Republic	63	Male	Homeless, alcoholism, smoking, myiasis, pediculosis of the head, burn wound infection	Yes	(33)
28	2021	Gmunden, Austria	79	Female	Smoking, terminally ill cancer, lung cancer, malnutrition (fatal)	No	(34)
29	2021	Baltimore, USA	63	Male	Homeless, deep vein thrombosis, chronic venous insufficiency, septic shock	Yes	(35)
30	2021	Dresden, Germany	90	Male	Tumorous skin formation (head, neck)	-	(2, 36)
31	2021	Dresden, Germany	82	Male	Renal failure, ulcus cruris	-	(2, 36)
32	2021	Dresden, Germany	79	Female	Diabetic foot, MRSA screening	-	(2, 36)
33	2021	Dresden, Germany	43	Male	Diabetic foot, ulcus cruris	-	(2, 36)
34	2021	Dresden, Germany	78	Male	Diabetic foot	-	(2, 36)
35	2021	Dresden, Germany	71	Female	Diabetic foot	-	(2, 36)
36	2021	Dresden, Germany	60	Male	Diabetic foot	-	(2, 36)
37	2021	Dresden, Germany	65	Male	Diabetic foot	-	(2, 36)
38	2021	Dresden, Germany	75	Male	Diabetes type 2	-	(2, 36)
39	2021	Dresden, Germany	43	Male	-	-	(2, 36)
40	2022	Baltimore, USA	48	Male	Smoking, diabetes type 2, right-foot plantar surface wound, osteomyelitis, deep soft tissue abscesses and tissue necrosis	Yes	(3)
41	2022	Ankara, Turkey	57	Male	10-year ex-smoker with a 30-year smoking history, soft tissue infection, osteomyelitis, rheumatoid arthritis	-	(37)
42	2022	Seoul, Korea	76	Male	Smoking, hypertension, diabetes, diabetic gangrene, ulcers	No	(38)
43	2022	Tulsa, USA	37	Male	Diabetes, chronic foot wound, osteomyelitis	No	(39)
44	2023	Royal Oak, USA	60	Female	Alcoholism, smoking, liver cirrhosis, peripheral vascular disease, chronic venous insufficiency, protein-calorie malnutrition	Yes	(40)
45	2023	Mechelen, Belgium	53	Male	Smoking, morbid obesity, incomplete spinal injury induced hemiparesis, chronic foot ulcer, osteomyelitis	Yes	(41)
46	2023	Kampala, Uganda	43	Female	-	-	(42)
47	2023	Guangzhou, China	50	Male	Diabetes, necrotizing fasciitis of left lower limb, wound infection-induced septic shock (fatal)	No	(12)

^*a*^ -, not mentioned.

**TABLE 2:** Overview of animal and other cases of *W. chitiniclastica*

Case	Year	Region	Source	Literature
1	2013	Janardhanapuram, India	Fish	(43)
2	2014	Michigan, USA	Deer	(44)
3	2015	Las Palmas de Gran Canaria, Spain	Dolphin	(45)
4	2015	Egypt	Aquatic plants	(18)
5	2015	China	Soils	(18)
6	2016	Charvadrason, Bangladesh	Arsenic-affected soils	(46)
7	2016,2023	Shenzhen, China	Zebra	(47, 48)
8	2016	Jinan, China	Dairy cow	(49)
9	2016,2019	Rio de Janeiro city, Brazil	Retail frozen chicken	(50, 51)
10	2017	Jilin, China	Cattle	(14)
11	2017	Korea	Soft-shelled turtles	(52)
12	2017	Knoxville, USA	Human gut microbiome of deceased individuals	(53)
13	2018	Mumbai, India	Retail marine fish	(54)
14	2021	California, USA	Vulture	(55)
15	2021	Jiangsu, China	Dairy cow	(15)
16	2022	Haozhou, China	Goat	(16)
17	2022	Khartoum, Sudan	Fermented animal and fish-based foods	(56)
18	2023	Noakhali, Bangladesh	Poultry chickens	(57)
19	2023	Anand, India	Retail chicken meat	(58)
20	2023	Sivas, Turkey	Dairy cow	(59)

The human cases of *W. chitiniclastica* were associated with bacteremia, myiasis, sepsis, cellulitis, osteomyelitis, ulcer, gangrene, soft tissue infection, and open wound infection. The risk factors include poor hygienic conditions, alcoholism, cardiovascular disease, chronic wounds/diseases, etc. (2, 25, 36). An important source of *W. chitiniclastica* is flies, such as *Wohlfahrtia magnifica* (1), *Lucilia sericata* (19, 28, 60), *Lucilia illustris* (61), *Chrysomya megacephala* (18, 62), *Hermetia illucens* (63), and *Musca domestica* (64). Around one-third of the cases have maggots (larvae) present (2). *W. chitiniclastica* might be attributed to the incidental contact between the open wound of the patient and flies (11, 26, 34) or the potential other insect transmission (2). On the other hand, a total of 16 cases have been linked to diabetes as of May 2024. It seems to suggest the diabetes mellitus could be a risk factor. More detailed studies are still required.

The animal reports related to *W. chitiniclastica* include fish (43), deer (44), dolphin (45), cattle (14), dairy cows (15, 49, 59), zebra (47), vulture (55), poultry chickens (57), soft-shelled turtles (52), and goat (16). Besides, there were several identifications of *W. chitiniclastica* from other special sources: aquatic plants (18), retail chicken meats (58), frozen chicken (50), retail marine fish (54), fermented animal- and fish-based foods (56), human gut microbiome of deceased individuals (53), soils (18), and arsenic-affected soils (46). It is worth mentioning that a case of swine-derived *W. populi* has been reported in China recently (65).

## IDENTIFICATION OF *W. CHITINICLASTICA*

*W. chitiniclastica* could be easily misidentified as other bacteria by conventional methods (general biochemical and morphological) due to the limitation of the databases. The misidentification of the documented reports was summarized in Table 3. The VITEK 2 system was unable to identify (13) or misidentify the strains as *Comamonas testosterone* (18), *Neisseria animaloris/zoodegmatis* (24, 30), *Acinetobacter lwoffii* (17, 21, 25, 49), or *Rhizobium radiobacter* (25). The API 20NE system may misidentify *W. chitiniclastica* as *A. lwoffi* (21), *Brevundimonas diminuta* (9, 21, 24, 45), or *Oligella urethralis* (9, 24). One case reported that MicroScan WalkAway system was unable to identify the organism, either (low probability scores for *Moxarella*/*Psychrobacter* spp., *Vibrio* spp., *Pasteurella*/*Actinobacillus* spp., and *Pseudomonas* spp.) (11). 16S rRNA gene sequencing and MALDI-TOF-MS are effective methods to detect *W. chitiniclastica*. With the more and more whole-genome sequencing of *W. chitiniclastica* has been made (41, 47, 48, 51, 62, 66), 16S rRNA sequencing can identify *W. chitiniclastica* at a very high level of confidence. On the other hand, compared with the expensive and cumbersome 16S rRNA, MALDI-TOF-MS technique could be a more suitable alternative to conduct easy, rapid, and accurate identification of *W. chitiniclastica* in clinical practice. With the development of science and technology, it has become the mainstream bacteria identification technique in recent years.

**TABLE 3:** Documented misidentification of *W. chitiniclastica*

Case	Year	Identification system	Misidentification of bacteria	Literature
1	2013	VITEK 2	Undefined	(13)
2	2015	VITEK 2	*Comamonas testosteroni*	(18)
3	2017/2020	VITEK 2	*Neisseria animaloris/zoodegmatis*	(24, 30)
4	2015/2019/2017 /2016	VITEK 2	*Acinetobacter lwoffii*	(17, 21, 25, 49)
5	2017	VITEK 2	*Rhizobium radiobacter*	(25)
6	2011/2017/2015	API 20NE	*Brevundimonas diminuta*	(9, 24, 45)
7	2011/2017	API 20NE	*Oligella urethralis*	(9, 24)
8	2017	MicroScan WalkAway	*Moxarella*/*Psychrobacter* spp., *Vibrio* spp., *Pasteurella*/*Actinobacillus* spp., and *Pseudomonas* spp.	(11)

*W. chitiniclastica* was often reported as part of the polymicrobial infection. For human cases, it has been identified together with *Myroides odoratimimus* (18, 25), *P. vulgaris* (21, 25, 36), *Klebsiella pneumoniae* (21), *A. lwoffii* (21), *Staphylococcus aureus* (21, 30, 33, 38), *Escherichia coli* (10, 25), *Aeromonas* spp. (10), *Streptococcus simulans* (10), *Bacteroides fragilis* (10, 27, 36), *P. mirabilis* (11, 25, 27), *Morganella morganii* (25, 27, 29, 36), *Serratia marcescens* (25), *Pseudomonas aeruginosa* (25), and *Providencia stuartii* (25, 28). In other reports, the *W. chitiniclastica* related polymicrobial infection occurred with *Myroides injenensis* (11), *Enterococcus faecalis* (11, 12), *Streptococcus anginosus* (27), *Streptococcus agalactiae* (27), *Ignatzschineria indica* (28, 30), *Providencia rettgeri* (8, 36), *Streptococcus pyogenes* (33), *Pasteurella canis* (41), *Streptococcus dysgalactiae* (37), *Arcanobacterium haemolyticum* (37), and *Streptococcus uberis* (17). However, it remains unclear if *W. chitiniclastica* was the real disease-causing agent in the polymicrobial infection.

## ANTIMICROBIAL SUSCEPTIBILITY PROFILE

*W. chitiniclastica* was reported to be susceptible to majority of known antibiotics, but intrinsically resistant to fosfomycin. However, the resistance mechanism of fosfomycin is still unclear currently and needs further study. In previous reports, *W. chitiniclastica* was susceptible to beta-lactams, quinolones, aminoglycosides, sulfonamides, carbapenems, and tetracyclines. An overview of the documented antibiotic susceptibility tests of *W. chitiniclastica* was obtained in Table 4. One can see that, in some cases, some drug resistances have emerged. This growing resistance should be a cause for concern. Beta-lactam (e.g*.*, ceftriaxone and penicillin) and quinolone (e.g*.*, ciprofloxacin and levofloxacin) antibiotics were used as empirical treatment in most cases. In the monomicrobial infection reports, ceftriaxone (7, 23), cefoperazone (24), cefoperazone/sulbactam (20), and cefazolin (40) showed good efficacy. On the other hand, there has been a case from China showing resistance to levofloxacin (12). A case showed that *W. chitiniclastica* was susceptible to norfloxacin. However, it was from a bovine. The human treatment cases of norfloxacin are lacking. Therefore, levofloxacin is still a good choice for the quinolone therapy (2). In general, although some resistances have been observed, beta-lactams and quinolones are currently safe for clinical use and should therefore be the recommended therapy.

**TABLE 4:** Results of the documented antibiotic susceptibility tests of *W. chitiniclastica*

Antimicrobial class	Antimicrobial agent	Susceptibility^*a*^	Literature
Beta-lactam	Amoxicillin	S	(7, 9, 13, 29)
	Ceftriaxone	S	(7, 9, 12, 16, 41)
	Imipenem	S	(7, 9, 12, 13, 17, 18, 20, 21, 24, 25, 27, 30, 33, 34, 36–38)
	Imipenem/cilastatin	S	(27)
	Penicillin	S	(9, 10, 37)
	Piperacillin	S	(9, 13, 20, 27, 36, 38)
	Piperacillin/tazobactam	S	(3, 11, 17, 18, 25, 27, 30, 31, 33, 34, 36)
	Ceftazidime	S	(3, 9, 11, 12, 17, 18, 20, 25, 27, 30, 33, 36, 38, 40, 41)
	Cefepime	S	(3, 11, 17, 18, 20, 21, 25, 27, 30–34, 36, 38, 40, 41)
	Amoxicillin/clavulanate	S	(20, 24, 33, 34, 41)
	Cefoxitin	S	(20)
	Ampicillin/sulbactam	S	(21, 36)
	Cephalosporin	S	(10)
	Ampicillin	S	(14, 24, 25, 36, 41)
	Cefotaxime	S	(24, 33, 34, 41)
	Cefoperazone	S	(24)
	Aztreonam	S	(17, 25, 27, 30, 36)
	Seond- and third-generation cephalosporins	S	(29)
	Sefepim	S	(37)
	Cefuroxime	S	(36, 41)
	Oxacillin	S	(14)
	Cefotaxime	I	(16)
	Ceftiofur	I	(16)
			
	Cefuroxime	R	(24)
	Piperacillin/tazobactam	R	(24, 32, 40)
	Penicillin	R	(12, 14–16)
	Ampicillin	R	(12, 16)
	Cefradine	R	(14, 16)
	Amoxicillin/clavulanate	R	(15)
	Cefalexin	R	(15)
	Amoxicillin	R	(16)
	Oxacillin	R	(16)
	Cefaclor	R	(16)
Quinolone	Ciprofloxacin	S	(3, 7, 9, 11, 14, 15, 17, 18, 20, 21, 25, 27, 32–34, 36, 38, 40)
	Levofloxacin	S	(11, 16–18, 21, 27, 30, 33, 36–38)
	Ofloxacin	S	(14, 33, 36)
	Moxifloxacin	S	(25, 36, 37)
	Norfloxacin	S	(14)
	Enrofloxacin	I	(16)
	Levofloxacin	R	(12)
	Ciprofloxacin	R	(24, 41)
	Moxifloxacin	R	(36, 37)
	Ofloxacin	R	(36)
Aminoglycoside	Amikacin	S	(3, 7, 11, 14, 15, 17, 18, 24, 27, 41)
	Gentamicin	S	(3, 9, 11, 13, 15, 17, 18, 20, 21, 27, 29, 30, 32, 36, 38, 40)
	Aminoglycoside	S	(10)
	Tobramycin	S	(3, 11, 17, 27, 30, 32, 34, 40)
	Neomycin	S	(14)
	Amikacin	I	(16)
	Gentamicin	I	(16)
	Amikacin	R	(36)
	Gentamicin	R	(24, 36, 41)
	Tobramycin	R	(12, 36)
	Streptomycin	R	(15, 16)
	Spectinomycin	R	(16)
Sulfonamide	Trimethoprim/sulfamethoxazole	S	(3, 7, 9, 11, 13, 14, 17, 18, 20, 21, 27, 29–32, 38)
	Trimethoprim/sulfamethoxazole	R	(15, 16, 41)
	Trimethoprim	R	(16)
Carbapenem	Meropenem	S	(3, 9, 12, 17, 18, 24, 27, 29, 33, 36, 41)
	Ertapenem	S	(12, 36)
	Carbapenem	S	(10)
	Doripenem	S	(36)
Tetracycline	Tetracycline	S	(9, 10, 13, 14, 37)
	Minocycline	S	(9, 17, 27)
	Doxycycline	S	(14)
	Doxycycline	I	(16)
	Tetracycline	R	(15, 30)
	Doxycycline	R	(15)
Polypeptide	Colistin	S	(17, 18)
	Vancomycin	S	(14, 37)
	Polymyxin B	S	(14)
	Polymyxin B	I	(16)
Fosfomycin	Fosfomycin	R	(12, 17, 41)
Macrolide	Erythromycin	S	(37)
	Azithromycin	I	(16)
	Erythromycin	R	(14)
	Roxithromycin	R	(16)
Lincosamide	Clindamycin	S	(14, 37)
	Clindamycin	I	(15)
	Clindamycin	R	(16)
Oxazolidinone	Linezolid	S	(37)
	Linezolid	R	(12)
Glycylcycline	Tigecycline	S	(12, 24, 36)
	Tigecycline	R	(36)
Nitrofuran	Furazolidone	S	(14)
Chloramphenicol	Chloramphenicol	S	(14)
	Flufenicol	R	(16)
Rifamycin	Rifampin	R	(16)

^*a*^ S, susceptible; I, intermediate; R, resistant.

## SUMMARY

In summary, *W. chitiniclastica* is a facultatively anaerobic, oxidase-positive, catalase-positive, Gram-negative, non-motile, and non-spore-forming *Gammaproteobacteria*. Recently, it has been described as a rare and emerging zoonotic pathogen. However, on the one hand, because of the diagnostic limitations of conventional biochemical and morphological methods, the bacterium might be misidentified. On the other hand, the local case reports often lack the international attention due to the language barriers. Therefore, *W. chitiniclastica* is insufficiently identified and underestimated. It is foreseeable that *W. chitiniclastica* will be more widely spread and identified with increased global communication and the advances in medical identification technology. The choice of the correct identification method should be paid attention to. MALDI-TOF-MS and 16S rRNA sequencing could serve as rapid and effective identification methods. Beta-lactam drugs and quinolones are recommended in the clinical treatment. In this work, we review the currently available literature. In particular, we have added previously overlooked cases from Chinese and other medical communities. We summarize the most complete cases of *W. chitiniclastica* from human, animal, and other sources. This work provides a complementary review of the previous work and would benefit future research.
